# Consumption of Raw Herbal Medicines Is Associated with Major Public Health Risks amongst Ugandans

**DOI:** 10.1155/2020/8516105

**Published:** 2020-06-03

**Authors:** Fred Ssempijja, Keneth Iceland Kasozi, Ejike Daniel Eze, Andrew Tamale, Sylvia Anurika Ewuzie, Kevin Matama, Justine Ekou, Paul Bogere, Regan Mujinya, Grace Henry Musoke, Jovile Kasande Atusiimirwe, Gerald Zirintunda, Muhamudu Kalange, Joel Lyada, Ritah Kiconco, Theophilus Pius, Christopher Nandala, Roland Mugisha Kamugisha, Yunusu Hamira, Edgar Mario Fernandez, Simon Peter Musinguzi

**Affiliations:** ^1^Department of Anatomy, Faculty of Biomedical Sciences, Kampala International University Western Campus, Box 71, Bushenyi, Uganda; ^2^Department of Physiology, Faculty of Biomedical Sciences, Kampala International University Western Campus, Box 71, Bushenyi, Uganda; ^3^Department of Physiology, School of Medicine, Kabale University, Box 317, Kabale, Uganda; ^4^Department of Wildlife and Aquatic Resources, School of Veterinary Medicine, College of Veterinary Medicine and Biosecurity, Makerere University, Box 7062, Kampala, Uganda; ^5^Department of Public Health, School of Allied Health, Kampala International University Western Campus, Box 71, Bushenyi, Uganda; ^6^Department of Clinical Pharmacy and Pharmacy Practice, School of Pharmacy, Kampala International University Western Campus, Box 71, Bushenyi, Uganda; ^7^Department of Animal Production, Faculty of Agriculture and Animal Sciences, Busitema University, Arapai Campus, Box 203, Soroti, Uganda; ^8^Faculty of Science and Technology, Cavendish University, Box 33145, Kampala, Uganda; ^9^Department of Medical Laboratory Sciences, School of Allied Health, Kampala International University Western Campus, Box 71, Bushenyi, Uganda; ^10^Department of Microbiology and Immunology, School of Medicine, Kabale University, Box 317, Kabale, Uganda

## Abstract

**Background:**

Community consumption of herbal plants in developing countries is a common practice, however, scarcity of information on their physiochemical composition is a major public health concern. In Uganda, *Vernonia amygdalina* is of interest in rural communities due to its therapeutical action on both bacterial and protozoal parasites, however no studies have been conducted to assess the heavy metal concentrations in traditional plants used in alternative medicine. The aim of the study was to establish concentrations of heavy metals in *Vernonia amygdalina*, model the estimated daily intake (EDI), and assess both the non-cancer-related health risk using the target hazard quotient (THQ), and the risk related to cancer through the incremental lifetime cancer risk (ILCR) for the Ugandan population.

**Methods:**

Leaves of *Vernonia amygdalina* were collected from 20 georeferenced villages and processed into powder in the laboratory using standard methods. These were then analyzed in the laboratory using an atomic absorption spectrometer for lead (Pb), chromium (Cr), copper (Cu), zinc (Zn), cobalt (Co), iron (Fe), cadmium (Cd), and nickel (Ni). Concentrations were compared against the World Health Organization (WHO) limits. The EDI, THQ, and ILCR were modelled and significance was measured at 95% confidence.

**Results:**

The study showed that mean ± SEM concentrations of heavy metals were highest in the order of Cr, 121.8 ± 4.291 ppm > Ni, 84.09 ± 2.725 ppm > Zn, 53.87 ± 2.277 ppm > Pb, 40.61 ± 3.891 ppm > Cu, 28.75 ± 2.202 ppm > Fe, 14.15 ± 0.7271 ppm > Co, 7.923 ± 0.7674 ppm > Cd, 0.1163 ± 0.005714 ppm. Concentrations of Pb, Cr, Zn, Co, and Ni were significantly higher than the WHO limits. The EDI was significantly higher in children than in adults, demonstrating an increased risk of toxicity in children. The THQ and ILCR were over 1000 times higher in all Ugandans, demonstrating the undesirable health risks following oral consumption of *Vernonia amygdalina* due to very high Cr and Ni toxicities, respectively.

**Conclusion:**

Consumption of raw *Vernonia amygdalina* was associated with a high carcinogenic risk, demonstrating a need to enact policies to promote physiochemical screening of herbal medicines used in developing countries against toxic compounds.

## 1. Introduction

Herbal medicines are currently used widely in complementary and alternative medicine for the management of various forms of ailments, and their diversity is important in developing communities [1]. Most herbal medicines are only used with the application of basic indigenous knowledge by the local community who lack pharmacological knowledge and this predisposes locals to toxic effects arising from unknown harmful elements in the plants [2]. The traditional plant *Vernonia amygdalina* is widely used in local communities of Africa (including Uganda) for home-based treatment of noncancer health conditions against bacterial and protozoal infections [3, 4], but detailed pharmacological knowledge and information on the presence of elements that could be toxic to humans is scarce. The most notable elements present in most medicinal herbs and vegetables are heavy metals such as lead (Pb), copper (Cu), nickel (Ni), iron (Fe), cobalt (Co), zinc (Zn), chromium (Cr), and cadmium (Cd) [5]. Essential elements include Co, Cu, Fe, and Zn due to their nutritional advantages in the body while nonessential elements include Ni, Pb, Cd, and Cr due to their carcinogenic effects [6].

Fe is an essential element important in a wide variety of metabolic processes, including oxygen transport, DNA synthesis, and electron transport [7]. Fe is required for the production of red blood cells and forms part of hemoglobin, helping in the binding and transportation of oxygen in the body [8]. Both Fe and Cu are important in oxygen and electron transport [9], demonstrating their synergistic effect in body physiology [10]. Fe toxicity leads to vomiting, diarrhea, and gastrointestinal tract (GIT) bleeding, shock, lethargy, hepatic necrosis, tachycardia, and metabolic acidosis and may sometimes lead to death, and prolonged exposure to high concentrations of Fe increases the risk of chronic ulcers and this predisposes one to gastric cancer [11]. Cu is an essential trace element playing an important role in human metabolism, primarily as a cofactor of many metalloenzymes helping the body to form red blood cells and maintain healthy bones, blood vessels, nerves, immune function, and it contributes to iron absorption and spermatogenesis [12–14]. Oral copper poisoning causes vomiting, hematemesis, hypotension, melena, coma, jaundice, and superficial or deep ulcerations of gastric and intestinal mucosa while in the liver, it causes dilatation of central veins, and in the kidneys, it leads to congestion of glomeruli and necrosis of tubular cells [14, 15]. Zn is an important micronutrient and essential as a catalyst and plays a role in structural, and regulation of antioxidant activity [16]. Zinc-binding motifs are found in many proteins encoded by the human genome physiologically, and free zinc is mainly regulated at the single-cell level [17]. Zn has a critical effect in homeostasis, immune function, oxidative stress, and apoptosis, and aging and significant disorders of great public health interest are associated with zinc deficiency [17]. Excessive absorption of Zn can suppress Cu and Fe absorption leading to deficiency of these elements. Oral intake of extremely high doses of zinc causes GIT symptoms such as nausea, vomiting, pain, cramps, and diarrhea. Chronic toxicity with Zn can cause alterations of blood lipoprotein levels, increased levels of low-density lipoproteins, and decreased levels of high-density lipoproteins [18]. Co is a relatively rare element, an essential nutrient to mammals in the form of cobalamin, i.e., vitamin B12 [18]. An adult human body contains about 1 mg of cobalt (total body content of Co is estimated at 1.1 and 1.5 mg) and 85% is in the form of vitamin B12 [19]. This directly implies it is important in the management of anemia associated with malabsorption of vitamin B12 in gastric ulcers [20]. Co toxicity mainly leads to cardiovascular, hematological, neurological, and endocrine defects [21, 22].

Toxicity due to Pb exposure is commonly associated with GIT irritation and neurotoxicity in children and adults [23]. Acute exposure to Pb can cause headache, loss of appetite, abdominal pain, fatigue, sleeplessness, hallucinations, vertigo, renal dysfunction, hypertension, and arthritis while chronic exposure can result in birth defects, mental retardation, autism, psychosis, allergies, paralysis, weight loss, dyslexia, hyperactivity, muscular weakness, kidney damage, brain damage, coma and may even cause death, and recently, it has been classified as a potentially carcinogenic compound [24, 25]. Cd leads to severe damages to the respiratory system and stomach irritation associated with nausea, vomiting, and diarrhea. Chronic-term exposure to Cd leads to its deposition in bones and lungs leading to bone and lung damage [26]. Cd is also highly toxic to the kidney causing renal dysfunction, and it is a carcinogenic agent known to induce lung and prostate cancer [27]. Cr is highly corrosive to the body, leading to allergic reactions such as urticaria [28, 29]. Ingestion of Cr can lead to anemia and cause damage to sperm and male reproductive system, GIT irritation, ulcers of the stomach and small intestines, cardiovascular, respiratory, renal, hepatic, and neurological effects, and death [30]. Chromium has been shown to promote carcinogenicity in stomach tumors [11, 31]. Nickel (Ni) is an essential element in some animal species, and it has been suggested to be an essential element for human nutrition [30, 32, 33], however, it has been strongly associated with dermatitis consisting of itching of the fingers, hands, and forearms, respiratory dystress in humans and an increased risk of lung and nasal cancers [34, 35]. It directly interferes with cell growth and has been implicated in genotoxicity, hematotoxicity, teratogenicity, immunotoxicity, and carcinogenicity [36, 37]. Mechanisms of heavy metal toxicity include the generation of free radicals to cause oxidative stress, damage of biological molecules such as enzymes, proteins, lipids, and nucleic acids, and damage of DNA leading to carcinogenesis [11]. Furthermore, noncancer degenerative disorders such as Parkinson's disease, multiple sclerosis, muscular dystrophy, and Alzheimer's disease have been linked to excessive consumption of heavy metals [11].

Heavy metals are measured to detect their concentrations in plant material to promote public health. When humans are exposed to moderate concentrations of these elements, either beneficial or toxic effects are inherently acquired, depending on their composition in the substance consumed. However, consumption above the safe or acceptable limits is associated with health risks due to heavy metal toxicity [2]. The permissible levels of heavy metals in medicinal plants set by the Joint Food and Agriculture Organization (FAO)-World Health Organization (WHO) committee for Fe, Cu, Cd, Pb, Cr, Co, Ni, and Zn are 20 ppm, 150 ppm, 0.3 ppm, 10 ppm, 2 ppm, 0.48 ppm, 2.14 ppm, and 27.4 ppm, respectively [5, 38]. Plants with higher concentrations of heavy metals above these limits are regarded as being unsafe for human consumption and pose major public health risks [2]. This was important since herbal plants are commonly used to manage various ailments in developing countries including Uganda [4, 39–42]. This may be an indicator of poor access to conventional healthcare [43], or chronic weaknesses in legislation to monitor herbal medicines in developing countries [44]. Most of the people in the herbal manufacturing and consumption industry rely on indigenous knowledge and lack access to information on pharmacodynamics and pharmacokinetics of most herbal therapies [41, 45, 46]. Studies in Uganda recommend that phytochemical investigations are necessary to determine active compounds in herbal medicines [4]; however, policy to support this remains weak. Most of the therapeutic uses of the documented herbal plants provide only basic data [1] with no conclusive toxicological analysis.

Self-medication usually involves combination of herbal plants with prescription medicine and this is influenced by indigenous knowledge on the plant [40]. In Uganda, knowledge on 39% of herbal plants used to treat malaria was from community and not scientific sources [41], demonstrating the importance of the current study. In Nigeria, drivers for self-medication have been associated with poor attitudes of healthcare workers and low efficacy of prescribed medications amongst undergraduate students [47], while in Egypt and Eretria, self-medication was common among the young, females, and being a medical student [48, 49], showing that medical students with medicines easily abuse the drugs at their disposal. Since traditional medicines are used heavily in developed and developing countries to manage infectious and noncommunicable diseases [39], consequences arising from potential adverse reactions and contraindications raise major policy challenges in the drug industry [42]. In India, very high concentrations of Pb and Cr in herbal plants rendered them unsafe [5]. Furthermore, in the United Arab Emirates, heavy metal concentrations in local and imported herbal plants rendered the plants unsafe for human use [50], showing a need for local authorities to routinely monitor the physicochemical composition of traditional plants in their communities.

WHO encourages extensive studies on herbal plants before they can be used in alternative medicine [51]; however, this is hardly followed in developing countries. This creates an atmosphere where quality assurance of herbal products and responsibility-sharing between the producer and the regulatory authorities through strict adherence to good agricultural collection practices (GACPs) and good manufacturing practices (GMPs) are impossible to effect [52]. In Kenya, weak legislation and regulation have created an ideal environment in which quacks practice, and this compromises drug safety and quality, justifying the need to enact clear and definitive legislation on herbal medicine use and practice [44]. In Uganda, the National Drug Authority (NDA) has not taken a keen interest in providing guidance, nor furthered research focused on pharmacological studies regarding these medicinal herbs [41], although these continue to be sold nationwide. Previously, we showed that community and commercial sources of drinking water are contaminated with heavy metals [53]; however, a scarcity of information from developing countries in Africa on the safety of herbal medicines justified this study. The objective of the study was to establish heavy metal concentrations in *Vernonia amygdalina* and model its public health safety for the Ugandan community where it is commonly used.

## 2. Methods

### 2.1. Study Design

This was an observational study conducted in Bushenyi district of southwestern Uganda as a follow-up to our previous study in the region [53]. A total of 20 plant samples were collected within the community. In each village, georeferenced coordinates (with an accuracy less than 3 m) were taken and used for mapping in an open-source software, i.e., quantum geographical systems (qGIS®) version 3.03 Cirona. Purposive sampling was conducted using community herbal medical attendants since these knew the major sources of *Vernonia amygdalina* in the community. The plant with its leaves was confirmed by a botanist at Kampala International University Western Campus. A total of 10 villages were selected, and in each, two samples were collected (*N* = 20).

### 2.2. Mapping of Survey Map for the Study Area in Southwestern Uganda

A Sentinel-2 image ID: L1C_TMRV_A022526_20191015T081349 with an acquisition date of 2019/10/15 from the United States Geographical Surveys (USGS) was used. This was superimposed on a shapefile for Uganda and roads. The satellite image file was modified to show 4 levels of classifications; band 1 (Red) was emphasized under single band pseudocolor continuous using settings in qGIS® which is an open-source software.

### 2.3. Preparation of Herbal Plant for Analysis

Leaves were washed using distilled water in the Department of Physiology and dried in an oven at 72°C for 2 days until the weight was constant. These were then grounded into powder using an electric mortar to gain fine powder and this was considered ready for laboratory analysis.

### 2.4. Preparation of Heavy Metal Standards

Working standard stock solutions for the heavy metals acquired from Germany were prepared as previously described [53]. Sample standards were prepared to make 0 parts per million (ppm), 0.5 ppm, 2 ppm, and 5 ppm, and their corresponding absorbance was determined using an atomic absorption spectrophotometer (AAS, PerkinElmer 2380), at a specific wavelength. Linear standard equations for each metal in the form *y* = *mx* + *c* were generated, where *y* = absorbance, *m* = gradient, *x* = concentration, and *c* = constant and *R*^*2*^*value* = level of accuracy; that is

  For Pb, wavelength of 217.0 nm, slit width of 1.0 nm, standard equation of *y* = 0.0168*x* + 0.0082, and *R*^2^ = 0.9763
  For Cr, wavelength of 357.9 nm, slit width of 0.2 nm, standard equation of *y* = 0.0193*x* + 0.0067, and *R*^2^ = 0.9792
  For Cu, wavelength of 324.9 nm, slit width of 0.5 nm, standard equation of *y* = 0.1152*x* + 0.0034, and *R*^2^ = 0.9996
  For Zn, wavelength of 213.9 nm, slit width of 1.0 nm, standard equation of *y* = 0.2051*x* + 0.1166, and *R*^2^ = 0.9209
  For Cd, wavelength of 228.8 nm, slit width of 0.5 nm, standard equation of *y* = 0.2075*x* + 0.0884, and *R*^2^ = 0.9559
  For Co, wavelength of 240.7 nm, slit width of 0.3 nm, standard equation of *y* = 0.0332*x* + 0.011, and *R*^2^ = 0.9842
  For Fe, wavelength of 248.3 nm, slit width of 0.2 nm, standard equation of *y* = 0.0304*x* + 0.0112, and *R*^2^ = 0.9815
  For Ni, wavelength of 232 nm, slit width of 0.2 nm, standard equation of *y* = 0.0362*x* + 0.0125, and *R*^2^ = 0.9836

### 2.5. Determination of Concentrations of Heavy Metals in *Vernonia amygdalina*

Concentrations of heavy metals were determined using standard methods [2, 54]. In 20 ml of nitric acid and 4 ml of analytical grade perchloric acid, 1 g of plant powder was added and allowed to mix and heated on a hot plate until the volume reached 4 ml. The solution was allowed to cool, it was filtered, and the final volume was adjusted by adding 50 ml of deionized water in the tubes. The heavy metals were then determined using AAS, Perkin Elmer, to generate absorbance at the corresponding wavelength for each sample. Using the standard equation (*y* = *mx* + *c*), the concentration for each sample was determined.

### 2.6. Modelling of Estimated Daily Intake of Heavy Metals in *Vernonia amygdalina*

EDI was determined using the following equation:

(1) $$\begin{array}{c} \text{EDI}=\frac{C\times \text{IR}}{\text{BW}}, \end{array}$$

where EDI = estimated daily intake, *C* = heavy metal concentration, and BW = body weight as previously described [53]. IR is the ingestion rate and this was 0.25 L/day and 0.75 L/day in Ugandan children and adults for *Vernonia amygdalina*, respectively [4]. Body weight for Ugandan children and adults of 15 kg and 70 kg, respectively, was used [53]. Children were defined as those aged between 5– 7 years and the mean age of 6 years was used, while their weight was 18–24 kg and the mean weight used was 20.5 kg which was in line with the Uganda National Guidelines [55]. Furthermore, adults are those 30 years [56], and this was in line with the Uganda National Guidelines for adults where a body weight greater than 50 kg is assigned to them [55].

### 2.7. Modelling of Noncancer Risk amongst Ugandans Associated with Consumption of *Vernonia amygdalina*

The target hazard quotient (THQ) was used to generate the hazard index (HI) to determine the presence of noncarcinogenic health effects following ingestion of the sampled water, i.e., THQ was determined for Pb, Cr, Cu, Zn, Cd, Co, Fe, and Ni using the following equation:

(2) $$\begin{array}{c} \text{THQ}=\frac{\text{CDI}}{\text{RfD}}, \end{array}$$

where CDI was the chronic daily intake for a particular metal obtained and RfD was the oral reference dose of the contaminant, i.e., RfD is an estimation of the maximum permissible risk on the human population through daily exposure.

(3) $$\begin{array}{c} \text{CDI}=\frac{\text{EDI }\times \text{EFr }\times \text{EDtot }}{\text{AT}}, \end{array}$$

where EDI is the estimated daily intake of a metal via ingestion of a specific route. EFr is the exposure (365 days/year). This is the process of estimating the intensity, frequency, and duration of human exposure to heavy metals via the oral route [57]. EDtot is the exposure duration (i.e. 6 years for children and 30 years for adults); and AT is the period of exposure for noncarcinogenic effects. EDtot is important since this preconditions for a hazard to have any effects in body tissues and this is influenced by the type of heavy metal and timing of exposure [58]. For noncancer risk modeling, AT = EFr × EDtot (2190 days in children and 10950 days in adults) and the reference dose (RfD) for each hazard was described previously [53], i.e., 0.004 ppm, 0.3 ppm, 0.001 ppm, 0.04 ppm, and 0.7 for Pb, Zn, Cd, Cu, and Fe, respectively. Exposure to multiple contaminants results in additive and interactive effects; thus, the hazard index (HI = ∑THQ) was used as an indication of risk [53].

### 2.8. Modelling of Cancer Risk amongst Ugandans Associated with the Consumption of *Vernonia amygdalina*

Following chronic exposure to carcinogenic heavy metals in the herbal plant, the incremental lifetime cancer risk (ILCR) was used to model the cancer risk in the Ugandan population using the following equation:

(4) $$\begin{array}{c} \text{ILCR}=\text{CDI}\times \text{CSF,} \end{array}$$

where CDI is the chronic daily intake of a particular metal and this was estimated over the 70-year lifespan for Ugandans (i.e., AT = 70 years × 365 days = 25550 days). In addition, the cancer slope factor (CSF) of Pb, Cr, Cd, and Ni of 0.0085 (ppm/day)^−1^, 0.5 (ppm/day)^−1^, 0.38 (ppm/day)^−1^, and 0.84 (ppm/day)^−1^ was used which is in line with US EPA limits [59–61].

### 2.9. Statistical Analysis

Data were cleaned in MS Excel version 2010, exported to GraphPad Prism version 6, and tested for normality by conducting the D'Agostino and Pearson omnibus normality test, and when *P* > 0.05, data were qualified for parametric tests. Information was presented as mean ± 95% CI and a one-sample *t*-test was conducted by making comparisons using the theoretical mean acquired from the WHO reference values and significance was reported when *P* < 0.05. A two-sample *t*-test was conducted to identify significant differences between children and adults on the estimated daily intake and cancer risk. Furthermore, the presence of cancer risk was identified when the ILCR was greater than 1 × 10^−4^. Superscript “a” was used for indication of risk while superscript “b” was used for the absence of risk in the Ugandan population.

## 3. Results

### 3.1. Description of the Study Area in Southwestern Uganda

**Figure 1:**
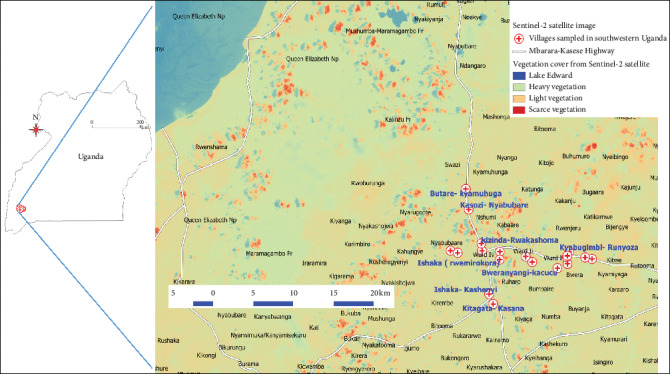
Sampled villages in the study area visualized on a Sentinel-2 satellite image file from USGS. Generally, the study area had moderate vegetation cover in comparison with the heavy vegetation associated with Queen Elizabeth National Park.

The study was conducted in village centers along the major highway of Mbarara-Kasese in Bushenyi district as shown in Figure 1.

### 3.2. Concentrations of Heavy Metals in *Vernonia amygdalina* in Uganda

The study showed that mean ± SEM levels of heavy metals were highest in the order of Cr > Ni > Zn > Pb > Cu > Fe > Co > Cd, i.e., 121.8 ± 4.291 ppm, 84.09 ± 2.725 ppm, 53.87 ± 2.277 ppm, 40.61 ± 3.891 ppm, 28.75 ± 2.202 ppm, 14.15 ± 0.7271 ppm, 7.923 ± 0.7674 ppm, and 0.1163 ± 0.005714 ppm, respectively. Concentrations of Pb, Cr, Zn, Co, and Ni were significantly higher, while concentrations of Cu, Cd, and Fe were found to be significantly lower than the WHO theoretical values as shown in Table 1.

### 3.3. Estimated Daily Intake of Heavy Metals in *Vernonia amygdalina* by Ugandans

Mean (±95% CI) estimated daily intake (EDI) of chromium was 2030.00 ± 149.17 mg/ml/day in children and 1305.00 ± 95.893 mg/ml/day in adults while for Pb, EDI was 676.83 ± 135.75 mg/ml/day and 435.1071 ± 87.2679 mg/ml/day for children and adults, respectively. EDI was highest in the order of Cr > Ni > Zn > Pb > Cu > Fe > Co > Cd and significant differences (*P* < 0.05) were observed amongst children and adults as shown in Table 2.

### 3.4. Noncancer Risk Associated with Consumption of Ugandan *Vernonia amygdalina*

Oral ingestion of *Vernonia amygdalina* was associated with a very strong noncancer risk due to significantly high Cr > Pb (greater than × 10^3^), followed by Ni > Cu (greater than × 10) in both children and adults. The noncancer risk was only absent in Fe for both children and adults (THQ < 1) as shown in Table 3.

### 3.5. Cancer Risk Associated with Consumption of Ugandan *Vernonia amygdalina*

Ingestion of *Vernonia amygdalina* was associated with a high risk of cancer and this was in the order of Ni > Cr > Pb > Cd. In adults, the risk of cancer was significantly (*P* < 0.05) higher than that in children, while in the latter, cancer risk was absent only in Cd. Furthermore, the cancer risk associated with the entire herbal plant was found to be over ×10^4^ above the recommended upper limit of ×10^−4^ in humans as shown in Table 4.

## 4. Discussion

The study was conducted along major towns in rural communities of southwestern Uganda (Figure 1). The resounding interest in this study area arises from the previous study conducted on environmental contamination of several water sources with heavy metals [53]. In this community, the use of herbal medicines was in agreement with related studies in central Uganda [4], demonstrating their importance to Ugandans. Heavy metals, i.e., Cu and Fe, which provide health benefits to humans, were found in significantly low concentrations when compared to the WHO limits for herbal medicines for human use (Table 1). These findings implied that the beneficial effects associated with Cu and Fe from *Vernonia amygdalina* in the oxygen and electron transport during metabolism [9] would be low in comparison to the very high concentrations of Pb, Cr, and Ni which have high carcinogenic potential and health risks, i.e., disruption of glucose utilization and initiation of apoptosis [31, 36]. In addition, concentrations of Co and Zn were high while the concentration of Cd was low in comparison with WHO limits on herbal medicines (Table 1), demonstrating the therapeutical effects of *Vernonia amygdalina* in Uganda on antioxidant activity and cellular metabolism, and properties associated with Co and Zn [16, 18] are stronger than those conferred by Cu and Fe. Current findings would help the Uganda National Drug Authority (NDA) to build an online archive of herbal medicines on the Ugandan market to protect the public and reverse the current situation where herbal medicines of unknown physiochemical composition are continuously being commercialized by the private industry [1, 4].

The estimated daily intake of heavy metals following oral consumption of *Vernonia amygdalina* by Ugandans was significantly highest in children than in adults (Table 2). This was because children have a relatively small body mass-to-volume ratio than adults, and thus, their rate of consumption is higher. This implied that the rate of exposure to heavy metals was also significantly greater in children than in adults, thus raising major public health concerns in the pediatric community. The World Health Organization (WHO) has set tolerable limits of heavy metals in herbal medicines, and once these are abused, toxic effects are bound to develop in humans [2, 5, 38]. This implies that NDA would take an active role and help streamline further studies in the country on the screening of herbal plants to ensure that medicinal therapies on the market are not associated with significant toxicological effects in the local population, thus helping shift the current deplorable status of nonsupervision of herbal enterprises in the country [41]. This would help promote good GACP and GMP in the herbal industry [52], for the promotion of public health.

The study showed that the risk of developing health conditions other than cancer was extremely high (i.e. above ×10^3^) due to excessive levels of Cr > Pb > Ni > Cu during the lifespan of children and adults (Table 3). These health conditions may include neurotoxicity, cell damage, and loss of cellular functions due to Cr and Ni through increased oxidative stress, DNA damage, and disruption of cellular signaling in the brain, lungs, liver, and kidney [11]. Furthermore, the risk of cancer was greatest in adults than in children (ILCR > ×10^−4^) due to high ILCR for Ni > Cr > Pb > Cd (Table 4). Nickel (Ni) is associated with dermatitis with lung and nasal cancers [34, 35] due to its ability to disrupt cellular growth [36, 37]. Cd leads to lung and prostate cancer [27] while Cr chronic ingestion leads to gastrointestinal cancers [11, 31]. These carcinogenic effects are common in adults than in children following chronic consumption of heavy metals. In this study, the carcinogenic threat of Ni was greater than that posed by Cr, thus showing that these heavy metals, although at low concentrations, i.e., Cd (Table 1), once accumulated over one's lifespan, can build up to increase the cancer risk in a population [6]. This was important since heavy metals cause oxidative stress in body tissues, leading to apoptosis and cellular dysfunction [11], demonstrating that concentrations of Ni raise major public health risks in herbal medicines. Furthermore, noncancer degenerative disorders such as Parkinson's disease, multiple sclerosis, muscular dystrophy, and Alzheimer's disease have been linked to excessive consumption of heavy metals [11]. Findings in this study demonstrate the risk posed by herbal medicines in Uganda and present a valid cause for the NDA to enact and spearhead policies that would help promote safe drugs including herbal plants in the general public.

## 5. Conclusion

The study showed that *Vernonia amygdalina* contains significantly high levels of carcinogenic compounds which predispose the local community to cancer over one's lifespan. Community consumption of raw herbal medicines should be discouraged by the authorities, and increased monitoring of herbal shops in Uganda and other developing countries should be promoted to reduce the disease risk. Persons interested in herbal therapies are advised to seek a conclusive diagnostic history on each plant before adding it on the list of alternative medicines for use. This would help reduce undue side effects which would haunt communities during their elderly period. Increased pharmacovigilance and training of team players in herbal medicines and research within Uganda would help policymakers enact practical policies to protect the public.

## Figures and Tables

**Table 1:** Description of heavy metal concentrations in *Vernonia amygdalina* and comparisons with WHO reference values on herbal plants for human consumption.

Variables (ppm)	Pb	Cr	Cu	Zn	Cd	Co	Fe	Ni
Number of values detected	20	20	20	20	6	20	20	20
Minimum	18.42	72.91	13.58	38.47	0.0960	2.970	7.750	53.26
Maximum	71.79	147.1	58.85	71.20	0.1320	14.47	19.39	101.7
Mean	40.61	121.8	28.75	53.87	0.1163	7.923	14.15	84.09
SEM	3.891	4.291	2.202	2.277	0.005714	0.7674	0.7271	2.725
Lower 95% CI of mean	32.46	112.8	24.14	49.10	0.1016	6.316	12.63	78.39
Upper 95% CI of mean	48.75	130.7	33.36	58.64	0.1310	9.529	15.67	89.79
WHO limits	10	2.0	150	27.4	0.3	0.48	20	10
*P* values	<0.0001	<0.0001	<0.0001	<0.0001	<0.0001	<0.0001	<0.0001	<0.0001
Summary	High	High	Low	High	Low	High	Low	High

SEM = standard error mean; CI = confidence interval; ppm = parts per million; Pb = lead; Cr = chromium; Cu = copper; Zn = zinc; Cd = cadmium; Co = cobalt; Fe = iron; Ni = nickel.

**Table 2:** Modeled estimated daily intake of heavy metals in *Vernonia amygdalina* in children and adults in Uganda.

Heavy metals detected	*N*	Children	Adults	*P* values
		Mean ± 95% confidence interval, estimated daily intake (mg/L/day = ppm/day)		
Pb	20	0.6768 ± 0.1358	0.43511 ± 0.08727	0.0036
Cr	20	2.030 ± 0.1492	1.305 ± 0.095893	<0.0001
Cu	20	0.4792 ± 0.07683	0.3080 ± 0.0494	0.0004
Zn	20	0.8978 ± 0.0795	0.5772 ± 0.05111	<0.0001
Cd	6	0.00194 ± 0.00025	0.001246 ± 0.000158	0.0003
Co	20	0.1321 ± 0.02678	0.08489 ± 0.017213	0.004
Fe	20	235.83 ± 25.33	0.1516 ± 0.01629	<0.0001
Ni	20	1.402 ± 0.095	0.9010 ± 0.0611	<0.0001

**Table 3:** Modeled noncancer health risks in Ugandans following consumption of *Vernonia amygdalina*.

Heavy metals detected	*N*	Children	Adults	*P* values
		Mean ± 95% confidence interval, target hazard quotient (THQ)		
Pb	20	193.38 ± 38.79^a^	124.32 ± 24.93^a^	0.0036
Cr	20	6766.67 ± 497.22^a^	4350.00 ± 319.64^a^	<0.0001
Cu	20	11.98 ± 1.92^a^	7.70 ± 1.23^a^	0.0004
Zn	20	2.99 ± 0.27^a^	1.92 ± 0.17^a^	<0.0001
Cd	6	3.88 ± 0.49^a^	2.49 ± 0.32^a^	0.0002
Co	20	4.40 ± 0.89^a^	2.83 ± 0.57^a^	0.0040
Fe	20	0.34 ± 0.036^b^	0.22 ± 0.023^b^	<0.0001
Ni	20	70.075 ± 4.75^a^	45.05 ± 3.054^a^	<0.0001
HI = ∑THQ		7053.71 ± 544.36^a^	4534.53 ± 349.95^a^	<0.0001

^a^Presence of noncancer risk and ^b^absence of noncancer risk.

**Table 4:** Modeled incremental lifetime cancer risk associated with *Vernonia amygdalina*.

Heavy metals detected	*N*	Children	Adults	*P* values
		Mean ± 95% confidence interval, ×10^−4^ incremental lifetime cancer risk (ILCR)		
Pb	20	4.93 ± 0.99^a^	15.85 ± 3.18^a^	<0.0001
Cr	20	870.00 ± 63.93^a^	2796.43 ± 205.48^a^	<0.0001
Cd	6	0.63 ± 0.0798^b^	2.03 ± 0.26^a^	<0.0001
Ni	20	1009.08 ± 68.40^a^	3243.47 ± 219.86^a^	<0.0001
∑ ILCR	66	1884.64 ± 133.40^a^	6057.78 ± 428.78^a^	<0.0001

^a^Presence of risk and ^b^absence of risk.

## Data Availability

Data files can be accessed at https://figshare.com/s/adc864290733ed7fc65a.
